# Effectiveness of Methylcobalamin and Folinic Acid Treatment on Adaptive Behavior in Children with Autistic Disorder Is Related to Glutathione Redox Status

**DOI:** 10.1155/2013/609705

**Published:** 2013-10-12

**Authors:** Richard E. Frye, Stepan Melnyk, George Fuchs, Tyra Reid, Stefanie Jernigan, Oleksandra Pavliv, Amanda Hubanks, David W. Gaylor, Laura Walters, S. Jill James

**Affiliations:** ^1^Department of Pediatrics, Arkansas Children's Hospital Research Institute, University of Arkansas for Medical Sciences, Little Rock, AR 72202, USA; ^2^Department of Biostatistics, Arkansas Children's Hospital Research Institute, University of Arkansas for Medical Sciences, Little Rock, AR 72202, USA

## Abstract

Treatments targeting metabolic abnormalities in children with autism are limited. Previously we reported that a nutritional treatment significantly improved glutathione metabolism in children with autistic disorder. In this study we evaluated changes in adaptive behaviors in this cohort and determined whether such changes are related to changes in glutathione metabolism. Thirty-seven children diagnosed with autistic disorder and abnormal glutathione and methylation metabolism were treated with twice weekly 75 *µ*g/Kg methylcobalamin and twice daily 400 *µ*g folinic acid for 3 months in an open-label fashion. The Vineland Adaptive Behavior Scale (VABS) and glutathione redox metabolites were measured at baseline and at the end of the treatment period. Over the treatment period, all VABS subscales significantly improved with an average effect size of 0.59, and an average improvement in skills of 7.7 months. A greater improvement in glutathione redox status was associated with a greater improvement in expressive communication, personal and domestic daily living skills, and interpersonal, play-leisure, and coping social skills. Age, gender, and history of regression did not influence treatment response. The significant behavioral improvements observed and the relationship between these improvements to glutathione redox status suggest that nutritional interventions targeting redox metabolism may benefit some children with autism.

## 1. Introduction

Autism is a neurodevelopmental disorder characterized by significant impairment in reciprocal social interaction and communication as well as restricted interests and repetitive behaviors. An estimated 1 of 88 individuals in the United States is affected with an autism spectrum disorder (ASD) [1]. Although several genetic syndromes are associated with ASD, all of these genetic syndromes together only account for a minority of ASD cases [2]. Other areas of novel research have concentrated on systemic physiological abnormalities, such as mitochondrial dysfunction [3, 4], oxidative stress [5–7], and inflammation/immune dysregulation [8–10]. These novel areas of research have substantially grown over the last decade [11]. These emerging areas of research have provided a new understanding of the diverse mechanisms involved in ASD and have promoted the idea that the autism spectrum is composed of several subgroups or endophenotypes [12]. 

Several lines of evidence support the notion of an ASD endophenotype with abnormal redox and methylation metabolism. In two case-control studies we reported that redox and methylation metabolism in children diagnosed with autism was abnormal compared to that of unaffected control children [6, 13]. Briefly, the mean ratio of plasma S-adenosyl methionine (SAM) to S-adenosyl homocysteine (SAH), a reflection of cellular methylation capacity, was significantly reduced. Also, the mean concentration of free reduced glutathione (GSH), the major intracellular antioxidant and mechanism for detoxification, was also significantly decreased and the oxidized disulfide form of glutathione (GSSG) was significantly increased. This imbalance in reduced as compared to oxidized glutathione resulted in a 2-fold reduction in the GSH/GSSG redox ratio, an index known as the glutathione redox status. Several precursors for methylation and glutathione synthesis were also lower in the autistic children suggesting an insufficiency in the production of methylation and glutathione metabolites, at least in a subgroup of ASD children. In addition, we have also demonstrated that GSH is significantly reduced in the cytosol and isolated mitochondria from lymphoblastoid cell lines and fresh peripheral blood mononuclear cells derived from children with autistic disorder (AD) [14, 15].

The glutathione provides the essential intracellular reducing environment required for normal immune function, detoxification capacity, redox-sensitive enzyme activity, and membrane redox signaling [16–20]. Oxidative stress occurs when antioxidant defense mechanisms fail to counterbalance reactive oxygen species generated from endogenous oxidative metabolism or from prooxidant environmental exposures. The notion that abnormal glutathione metabolism is associated with oxidative cellular damage is consistent with studies from our laboratory which demonstrate an increase in oxidative damage to protein and DNA in peripheral blood mononuclear cells and postmortem brain derived from ASD individuals [15, 21]. Several recent reviews and research studies also lend support to the hypothesis that redox imbalance and oxidative stress may be a contributing factor to autism pathophysiology [6, 22].

These findings have particular clinical relevance since abnormalities in redox metabolism are potentially amenable to treatment. Glutathione is a tripeptide of cysteine, glycine, and glutamate that is synthesized *de novo* in all cells. Glutathione synthesis is tightly connected to methylation metabolism where it derives its cysteine precursor. As both methylation and glutathione metabolism have been shown to be abnormal in children with ASD and due to the fact that abnormal methylation metabolism will result in a depletion of the precursors needed for glutathione production, we previously conducted an open-label intervention trial where targeted metabolic cofactors for methylation and glutathione metabolism were provided. Specifically, in our previous study, we determine if glutathione redox status could be improved with a three-month intervention of subcutaneously injected methylcobalamin and oral folinic acid in children with AD and metabolic evidence of abnormal methylation and glutathione metabolism [23]. Although we measured adaptive behavior using the Vineland Adaptive Behavior Scale (VABS) during the trial, this behavioral data has not been previously reported. We now report the change in communication, daily living, and social skills domains of the VABS associated with the three-month intervention as well as investigate whether improvement in glutathione redox status was related to any change in VABS behavior scores.

## 2. Methods and Materials

### 2.1. Participants

Forty-eight children with AD were enrolled in an open-label trial to test whether a three-month treatment of methylcobalamin (methyl B12) and folinic acid would improve glutathione synthesis as well as adaptive behavior. Inclusion criteria were a diagnosis of AD as defined by the *Diagnostic and Statistical Manual of Mental Disorders, Fourth Edition *(DSM-IV) criteria (299.0) and a Childhood Autism Rating Scales (CARS) score greater than 30. Exclusion criteria included Asperger's disorder, pervasive developmental disorder-not otherwise specified (PDD-NOS), genetic disorders, epilepsy, severe gastrointestinal symptoms, recent infections, and/or current use of high-dose vitamin or mineral supplements (i.e., above the recommend daily allowance). Metabolic and behavioral assessments were conducted at baseline and at the end of the 3-month intervention. A nurse contacted each family every two weeks to ensure compliance and monitor adverse effects.

**Figure 1:**
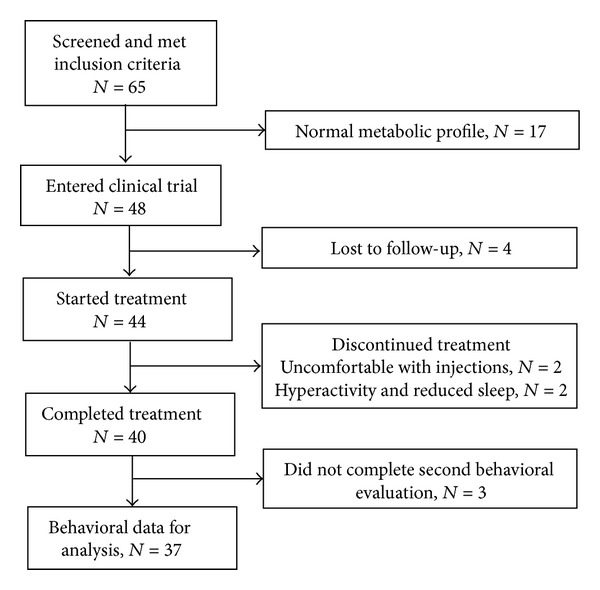
Flowchart of patients who met inclusion criteria for the study.

Figure 1 provides a flowchart of the patients who met criteria for possible inclusion in the study. In order to determine if abnormal redox metabolism could be improved with the intervention, we selected children with abnormal glutathione redox status and methylation capacity for entry into the study. Normal glutathione redox status and methylation capacity were defined by measuring these same metabolites in typically developing control children. Sixty-five autistic children met the inclusion criteria and were initially screened for metabolic evidence of abnormal methylation capacity (SAM/SAH < 3.0) and glutathione redox metabolism (GSH/GSSG < 6.0). Of these, 48 (75%) children were found to have both abnormal methylation capacity and glutathione redox metabolism, thus meeting metabolic qualifications for inclusion. The remaining 17 (25%) were excluded because their baseline metabolic profile was within normal range. Four children dropped out during the study and another four children were lost to follow-up. Two children dropped out of the study because the parents were uncomfortable giving the methylcobalamin injections and 2 children dropped out because of hyperactivity and reduced sleep. For the four that were lost to follow-up, every attempt was made to contact the family but they either did not response to such requests or moved out of the area and could not return for follow-up. Of the 40 remaining children who completed the study, three of the parents did not complete the second behavioral evaluation, leaving 37 children who completed both the intervention and the behavioral evaluation. Characteristics of the children that completed the study are given in Table 1. Adverse effects were minimal with occasional reports of hyperactivity, as summarized in our previous study [23] and below. All parents signed informed consent approved by the Institutional Review Board at the University of Arkansas for Medical Sciences.

### 2.2. Intervention

Methylcobalamin was provided as a sterile injectable liquid from Hopewell Compounding Pharmacy (Hopewell, NJ). Tuberculin syringes fitted with a 1/4 inch 31 gauge needle were prefilled with 75 *μ*g/Kg methylcobalamin and prepared individually based on each child's weight. A demonstration and instructions for the sterile subcutaneous injection of methylcobalamin in the fatty tissue of the buttocks were given to all parents. Parents were instructed to give methylcobalamin every third day for 3 months. Folinic acid (400 *μ*g) obtained from Custom Compounding (Little Rock, AR) was administered orally twice each day as a powder mixed with a convenient food. 

The subcutaneous injectable route of administration of methylcobalamin was selected based on preliminary observations that it resulted in improvement in speech and cognition [13] and the potential for support of methionine synthase activity under conditions of oxidative stress by substituting for the oxidized inactive form of coenzyme B12 [Cob (II)]. Folinic acid (5-formyltetrahydrofolate) was selected in light of the fact that it is absorbed as the reduced metabolite is rapidly polyglutamated and is more readily available for folate-dependent reactions than the synthetic vitamin form of folic acid. In our previous study, we demonstrated that this treatment significantly improved GSH and GSSG concentrations and glutathione redox status (i.e., the GSH/GSSG ratio) [23].

### 2.3. Glutathione Measurement

Fasting blood samples were collected into EDTA-Vacutainer tubes and immediately chilled on ice before centrifuging at 4000 ×g for 10 minutes at 4°C. To prevent metabolite interconversion, the ice-cold samples were centrifuged within 15 minutes of the blood collection and the plasma stored at −80°C until high-pressure liquid chromatography quantification within 2 weeks after receipt. Details of the methodology for high-pressure liquid chromatography with electrochemical detection and metabolite quantitation have been previously described [24, 25].

### 2.4. Behavioral Evaluation

The VABS (1st Edition) is a validated parent interview that provides a numerical score for adaptive functioning in the domains of communication, socialization, and daily living skills which has been shown to have good reliability and validity [26]. Nine subscales represented these three domains, specifically receptive, expressive, and writing communication, personal, domestic, and community daily living skills, and interpersonal relations, play-leisure, and coping skills. Raw scores were used in the statistical analysis while age equivalent scores are used to illustrate the magnitude of the changes in behavioral development over the treatment period and domain scaled scores are provided as a comparison of the participant sample to the general population. Higher raw, age equivalent, and scaled scores represented better performance on these measures. The VABS was administered by a trained nurse at the baseline visit and again at the end of the treatment period.

### 2.5. Statistics

Mixed-effects regression models were conducted via SAS version 9.3 (Cary, NC) “glmmix” procedure [27]. Raw scores were used in the statistical analysis to represent the change in behavior over the intervention period. A similar approach was used in our recent study on the effect of sapropterin treatment in children with ASD [28]. Raw scores were used due to the fact that transformation of raw scores into scaled scores is not statistically valid to follow changes in adaptive behavior especially when examining the moderating effect of a biomarker on the change in behavior and especially when there is no reference control group. For example, many children with ASD have slow or stagnant development, so their development may not change over a typical three-month interval. With an intervention, their development may approach the rate of a typically developing child. However, a child whose development changes over the three-month period at a rate equal to that of a typically developing child would have a stable unchanging scaled score. Thus, in such a case, the scaled score would appear to reflect no developmental gains over the treatment period even though the child was making developmental gains. Since the null hypothesis is that there is no change over the treatment period, an unchanging scaled score would not be statistically significant even though it would indicate developmental progress.

The first statistical model included a linear effect of time on raw scores. A random intercept was used to account for each individual's symptom level. Two-tailed alpha of 0.05 was used in all analyses. In these initial models we tested whether age, gender, and/or a history of regression influenced the change in the outcome measure. To illustrate the relative strength of the effect we provide the Cohen's *d* effect size for each significant change in outcome variables. Next we determined whether baseline and/or change in glutathione redox status (i.e., the free reduced-to-oxidized glutathione ratio) moderated changes in the outcome variables. The glutathione redox status was investigated because it significantly changed with treatment in our previous study [23]. We did not investigate the moderating effect of other metabolites as multiple statistical tests would have increased our type I error rate. We conducted two sets of analyses; one examined the moderating effect of the overall glutathione redox status and another that examined the moderating effect of the change in glutathione redox status over the treatment period. The former analysis was conducted to determine whether glutathione redox status in general was related to VABS scores, while the latter analysis was conducted to determine if the change in glutathione status was related to the change in VABS scores over the treatment period. Age, gender, and/or regression factors were included in the analyses examining the moderating effect of glutathione if they were significant in the initial model.

## 3. Results

### 3.1. Behavior Change over the Intervention Period

The VABS was used to examine the impact of the intervention on measures of core behaviors associated with autism. The changes in VABS subscales with treatment were not influenced by gender, age, or history of regression, and neither gender nor history of regression was related to overall VABS subscale scores. Age was significantly related to overall raw scores for all subscales, except for interpersonal and coping skills. 

Significant increases were found in all VABS subscale scores, including receptive (*F*(1,36) = 11.90, *P* = 0.001; *d* = 0.59), expressive (*F*(1,36) = 32.80, *P* < 0.0001; *d* = 0.97), and written (*F*(1,36) = 11.07,  *P* < 0.005;  *d* = 0.56) communication skills, personal (*F*(1,36) = 14.69, *P* < 0.0005; *d* = 0.65), domestic (*F*(1,36) = 4.85, *P* < 0.05; *d* = 0.37) and community (*F*(1,36) = 9.51, *P* < 0.005; *d* = 0.52) daily living skills, and interpersonal (*F*(1,36) = 6.45, *P* < 0.05; *d* = 0.43), play-leisure (*F*(1,36) = 12.36, *P* = 0.001; *d* = 0.59), and coping (*F*(1,36) = 15.68, *P* = 0.0005; *d* = 0.66) social skills. 

**Figure 2:**
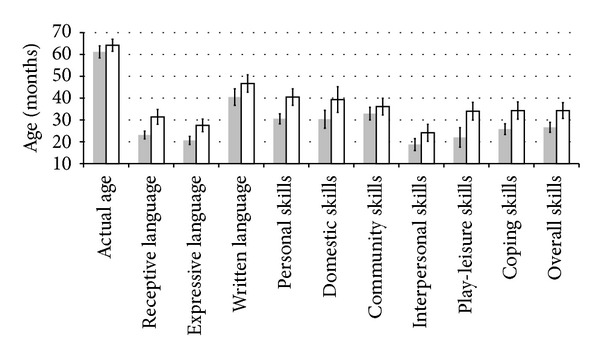
Histogram of actual age and age equivalents for the Vineland Adaptive Behavior Scale subscales. Error bars depict standard error.

To illustrate the magnitude of the change in development with the intervention, Table 2 and Figure 2 present the average age equivalent for all subscales before and after the intervention. Table 2 also demonstrates the change in age equivalent with the intervention including confidence intervals. As evident from this table, many subscales, particularly those with larger effect sizes, demonstrate large gains with the three-month intervention. This is particularly true for the communication domain where skills improved between 6.0 and 8.3 months, on average, and in the social skills domain where skills improved between 5.4 and 12.0 months, on average, over a three-month period.

Baseline pretreatment scores of the individual domains and overall composite of the VABS (mean (SD): communication 65.4 (13.0); daily living skills 66.4 (13.4); social skills 67.3 (9.3); adaptive behavioral composite 66.1 (9.3)) were found to be within the range of previously published VABS scores for children with autistic disorder [15, 29]. Following intervention all individual domains and the overall composite of the VABS markedly increased (Mean (SD): communication 72.1 (15.7); daily living skills 76.0 (18.0); social skills 75.6 (16.5); adaptive behavioral composite 73.8 (17.4)).

### 3.2. Relation between Behavior and Glutathione Redox Status

**Figure 3:**
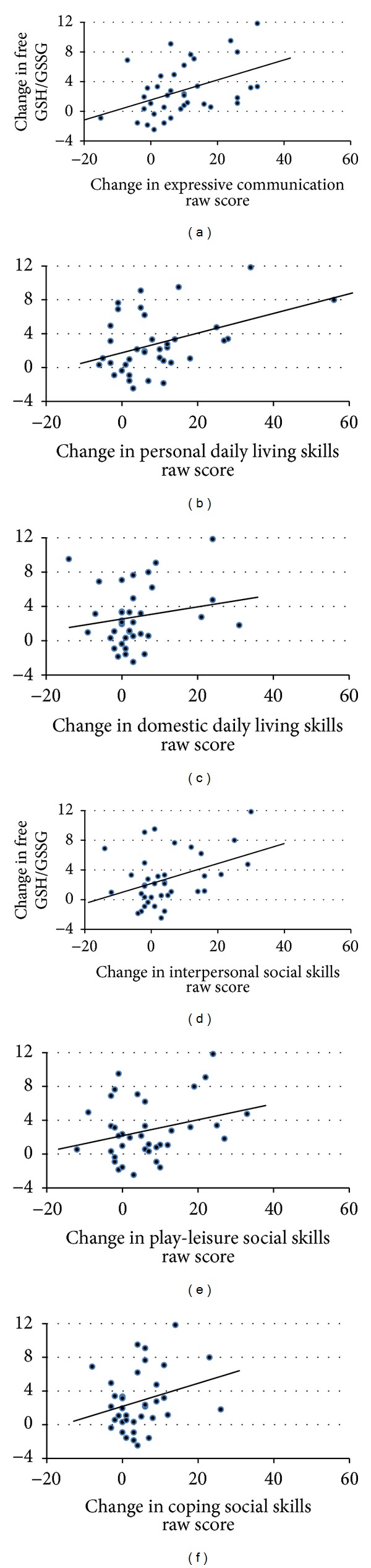
Significant relationships between the change in the glutathione redox status (reduced-to-oxidized glutathione ratio) and change in subscales of the Vineland Adaptive Behavior Scale (VABS) subscales. An improvement in glutathione redox status was associated with improvement in (a) expressive communication (*F*(1,33) = 9.66, *P* < 0.01), (b) personal daily living skills (*F*(1,34) = 12.84, *P* = 0.001), (c) domestic daily living skills (*F*(1,34) = 4.69, *P* < 0.05), (d) interpersonal social skills (*F*(1,34) = 10.47, *P* < 0.005), (e) play-leisure social skills (*F*(1,34) = 8.16, *P* < 0.01), and (f) coping social skills (*F*(1.34) = 6.09, *P* < 0.05). The moderating effect of glutathione redox status on the VABS subscale is provided in the graph as a representation of the relationship between the variables. Since the linear models examining the moderating effect of glutathione redox status on the VABS subscale takes into account age, simple correlation coefficients would not be accurate for inclusion in the graphs.

The overall glutathione redox status was not related to VABS subscales, indicating that overall development did not appear to be related to overall glutathione redox status. In order to determine whether changes in VABS subscales were related to changes in glutathione redox status, we examined whether the glutathione redox status moderated the change in the VABS subscales. We found that glutathione redox status moderated expressive communication (*F*(1,33) = 9.66, *P* < 0.01), personal (*F*(1,34) = 12.84, *P* = 0.001) and domestic (*F*(1,34) = 4.69, *P* < 0.05) daily living skills, and interpersonal (*F*(1,34) = 10.47, *P* < 0.005), play-leisure (*F*(1,34) = 8.16, *P* < 0.01), and coping (*F*(1,34) = 6.09, *P* < 0.05) social skills such that a greater increase in the glutathione redox status (i.e., greater improvement in glutathione) was associated with a greater improvement in VABS subscale scores (see Figure 3).

### 3.3. Adverse Effects

The adverse effect rates are outlined in Table 3. Out of the forty-four children that started the study and were not lost to follow-up, 72% reported no adverse effects of the treatment. For the four children that dropped out of the study two children (5%) dropped out because the parents were uncomfortable giving the methylcobalamin injections and 2 children (5%) dropped out because of hyperactivity and reduced sleep. Of the 4 families who reported hyperactivity but did not drop out of the study, the hyperactivity resolved with decreasing the folinic acid dose to 400 *μ*g per day.

## 4. Discussion

This intervention trial was undertaken to determine whether a nutritional intervention consisting of treatment with metabolic precursors for methionine and glutathione synthesis would improve autism symptoms in children with AD. Children meeting the inclusion criteria were initially screened for metabolic evidence of abnormal methylation capacity and glutathione redox status as an indication of potential benefit from the intervention. Our goal was to determine whether nutritional support with precursors for the abnormal metabolic pathways could result in improvement in ASD associated behavior as measured by the VABS. Overall, significant improvement was noted on all subscales of the VABS during the three-month intervention. In addition, improvement on several VABS subscales, including those related to communication and social skills, was related to the improvement in glutathione redox status. Overall, this study provides preliminary evidence that targeted nutritional support in children with AD who have metabolic abnormalities in glutathione and methylation pathways may be associated with improvements in certain behaviors associated with ASD, at least in a subset of AD children. In addition, a low rate of adverse effects with methylcobalamin and folinic acid treatment indicates that these supplements are safe and warrants further study as an intervention.

### 4.1. Behavioral Improvements with Intervention

A simple and safe nutritional intervention of methylcobalamin and folinic acid resulted in significant increases in VABS scores for all domains, including daily living, social, and communication skills, with an average effect size of 0.59, which is in the medium-to-large range. In fact, even the smallest effect size (domestic daily living skills, *d* = 0.37) was in the medium range and the largest effect size (expressive language skills, *d* = 0.97) was in the very large range. When considering changes in age equivalent skills over the treatment period, it was found that skills improved an average of 7.7 months over the three-month treatment period, with the lower end of the range being an average gain of 2.0 months for community daily living skills to the higher end of the range being an average gain of 12.0 months improvement for play-leisure skills. However, when we examine the overall change in scaled scores across the three-month treatment period, we find that, on average, the scaled score for the individual domains and the adaptive behavioral composite at the end of the treatment were still well below the average for typically developing children. In fact, these scaled scores were, on average, at the very lower end of normal range. Thus, although significantly improved, the behavior scores after treatment remained, on average, at the very low end of normal and for many children below the normal range. This suggests that further therapy may be required to promote continued improvement through continued nutritional treatment with metabolic precursors over a prolonged time period, the addition of intensive behavioral and educational therapy, or both. 

### 4.2. The Relationship between Metabolic and Behavioral Changes

Change in glutathione redox status with treatment, but not baseline glutathione redox status prior to treatment, was found to be related to improvements in several VABS subscales, including all of the subscales in the social skills domain, two of three subscales in the daily living skills domain, and one of the subscales in the communication domain. Interestingly, many of the VABS subscales that were related to glutathione redox status were also subscales that demonstrated large effect sizes and larger age equivalent gains (e.g., personal daily living skills and play-leisure and coping social skills). This suggests that the marked changes in behaviors observed in these particular subscales were not simply due to a placebo-effect and may indeed be related to improvements in glutathione redox status over the three-month treatment period, although we will have to await double-blind placebo-controlled studies to validate these findings. The lack of a relationship between overall glutathione redox status and VABS scores most likely reflects the preselection of children with low glutathione redox status, thus decreasing the overall range in glutathione redox status.

In our previous study, we demonstrated that this open-label intervention significantly improved metabolites of glutathione metabolism, including total and free GSH and GSSG concentrations and the glutathione redox status (i.e., the GSH/GSSG ratio) [23]. However, despite this improvement, total and free GSH concentrations and the glutathione redox status remained significantly below those of age-matched control children. Additionally, methionine, SAM, and SAH concentrations did not significantly change with the intervention despite the fact that both methylcobalamin and folinic acid provide methyl groups for the methionine cycle. The fact that the treatment improved but did not normalize methionine, SAM and glutathione concentrations may reflect ongoing metabolic compensation for incompletely resolved oxidative stress. Thus, continued prooxidant conditions may promote glutathione synthesis as the metabolic priority at the expense of methionine transmethylation. Treatment with methylcobalamin and folinic acid appears to have rescued glutathione synthesis in this cohort of children, albeit incompletely, at the expense of transmethylation metabolism. Given the fact that some behavioral improvements were related to improvements in glutathione status, it is possible that longer-term treatment or treatments that include additional precursors or methylcobalamin and folinic acid at higher doses may result in additional behavioral improvements. For example, one recent open-label study demonstrated that high-dose folinic acid demonstrated potential efficacy in children with ASD [30] and another double-blind placebo controlled study demonstrated that N-acetylcysteine, a glutathione precursor, resulted in decreased irritability and improved social function [31]. However, neither of these aforementioned studies measured redox metabolism in order to determine whether the observed behavioral effects were related to changes in redox metabolism. Further studies will be needed to determine the optimal combination of precursors for the treatment of redox abnormalities in children with ASD.

### 4.3. The Use of Novel Interventions in Autism Spectrum Disorder

Our findings are consistent with smaller [31, 32] and larger [33] randomized control trials and large case series [30] demonstrating that nutritional interventions for children with ASD that target oxidative stress are associated with improvements in core ASD symptoms [30, 31], sleep and gastrointestinal symptoms [32], and hyperactivity, tantruming and parental impression of general functioning [33]. Besides the methylcobalamin and folinic acid treatment used in this study [23], the beneficial effect of nutritional treatments on redox metabolism in children with ASD has been documented in a randomized control trial [33] and a cross-sectional study [34]. Such studies suggest that glutathione metabolism can be improved by vitamin and mineral [33] and antioxidants, coenzyme Q10 and B vitamins supplementation [34], and sapropterin treatment [28]. Thus, although there is evidence for nutritional treatments improving redox metabolism and ASD symptoms and behaviors in children with ASD, there is no specific established treatment for correcting redox abnormalities in children with ASD and the evidence for efficacy of nutritional treatments which address oxidative stress on core and associated ASD behaviors is preliminary. Clearly more studies are needed to clarify the potential beneficial effect of nutritional treatments for children with ASD.

The nutritional treatment used in this study is considered, by some, as a complementary and alternative medicine (CAM) treatment [35]. However, CAM treatments refer to a much wider variety of treatments, including nonnutritional treatments such as acupuncture and homeopathy. The prevalence of CAM in children diagnosed with a ASD in western countries is estimated to be between 32% and 87% [35] which is believed to be much higher than CAM therapies used in typically developing children [36]. Children with ASD with a greater number of medical and behavioral problems receive more CAM treatments than those with fewer medical and behavioral problems [37]. Parents believe that most CAM-associated treatments are either helpful or without effect but not harmful [35]. The main reasons parents cite for choosing to use CAM for ASD are concerns about the safety and side effects of available medications for ASD and a need to be involved in decisions involving care of their child [38]. Because the majority of CAM therapies are based on anecdotal evidence, there is a clear need for clinical trials to evaluate the efficacy of these treatments [35]. In addition, it should be noted that for ASD, of all treatments considered CAM, nutritional treatments have the most evidence for effectiveness and efficacy. Thus, it may not be proper to consider nutritional treatments, which have been used in mainstream medicine for over a century, as equivalent to CAM treatments which have undergone much fewer investigations and have less support for their use. 

### 4.4. Limitations

The reported improvement in behavior scores by parent report should be interpreted with caution in an open-label trial because of expectation bias [38]. In addition, without a control group it is difficult to know if, and to what extent, development would have changed without treatment. As this is a group with significant delays, it is unlikely that they would have demonstrated typical gains over the three-month period but it is possible they may have shown some developmental progress. However, it is reassuring that a significant number of improvements were moderated by improvements in glutathione metabolism, suggesting that changes in metabolism were indeed related to improvements in behavior. 

This study prescreened and selected a subgroup of children with ASD that demonstrated abnormal glutathione redox status and methylation capacity as a means to increase sensitivity to treatment. The broad heterogeneity of clinical and behavioral symptoms in autistic children predicts that no single treatment will benefit every autistic child. Thus, the definition and characterization of subgroups of children who respond positively or negatively to intervention will be necessary to more sensitively identify those children who are most likely to benefit from a given treatment or medication. 

### 4.5. Conclusions

This study demonstrates that a three-month treatment with methylcobalamin and folinic acid is associated with significant improvements in behavioral symptoms associated with ASD in a group of children with AD and metabolic markers of abnormal redox and methylation metabolism. Our previous study demonstrated that this treatment also improves metabolic markers of glutathione metabolism in these same children. This provides convergence of independent measures demonstrating the beneficial effect of this simple and safe nutritional intervention. Clearly this intervention requires further study in a double-blind placebo-controlled trial to eliminate the potential bias associated with an open-label study.

## Figures and Tables

**Table 1:** Characteristics of the 37 participants with autistic disorder who completed the trial.

Gender (% male)	81%
Age (mean ± standard deviation)	5.1 ± 1.4 years
Regression (% regression)	35%
Childhood Autism Rating Scale score (mean ± standard deviation)	39.2 ± 7.8

**Table 2:** Age equivalent scores from the Vineland Adaptive Behavior Scales at baseline before and after 3-month intervention with methylcobalamin and folinic acid. The change in age equivalent scores with 95% confidence interval (CI) is also given. The overall average of all subscales is also given in the last row of the table.

Vineland subscale	Baseline age equivalent (months) (mean ± SE)	Postintervention age equivalent (months) (mean ± SE)	Change in age equivalent (months) (mean; 95% CI)
Receptive language	23.1 ± 1.8	31.4 ± 3.4	8.3 (2.9, 13.7)
Expressive language	20.6 ± 1.9	27.5 ± 2.9	6.0 (3.3, 9.4)
Written language	40.5 ± 3.8	46.7 ± 4.0	6.2 (3.4, 9.0)
Personal skills	30.5 ± 2.3	40.5 ± 3.8	10.0 (3.8, 16.2)
Domestic skills	30.3 ± 4.1	39.3 ± 5.9	9.0 (−1.4, 19.4)
Community skills	32.9 ± 2.9	36.1 ± 3.8	2.0 (−3.0, 6.9)
Interpersonal skills	18.7 ± 2.7	24.1 ± 3.9	5.4 (0.0, 10.9)
Play/leisure skills	22.0 ± 4.5	34.0 ± 4.1	12.0 (4.1, 19.6)
Coping skills	25.8 ± 2.5	34.3 ± 4.0	11.5 (4.9, 18.0)
Overall skills	26.6 ± 2.3	34.3 ± 3.6	7.7 (3.4, 12.0)

**Table 3:** Adverse effects reported of intervention with methylcobalamin and folinic acid. All 44 children who entered the study but were not lost to follow-up were included.

Adverse effect	% (*N*)
Hyperactivity	14% (6)
Reduced sleep	7% (3)
Discomfort with injections	5% (2)
Insomnia	2% (1)
Impulsivity	2% (1)
Irritability	2% (1)
